# Supplementary material for: Predicting visual function by interpreting a neuronal wiring diagram
Source: Nature. 2024 Oct 2;634(8032):113–23. doi: 10.1038/s41586-024-07953-5 (PMC11446822; doi:10.1038/s41586-024-07953-5)
Supplement: Supplementary file 6 — CRF and ERF predictions for individual TmY4 and TmY9 cells. Analogous to Supplementary Data 3, but for TmY target types. Shown are the top four monosynaptic pathways, the strongest pathway passing through each of the top ten intermediary types (ranking from Extended Data Fig. 7), and the trisynaptic pathway Tm1–TmY–Dm3–TmY (see the section entitled Prediction of spatial normalization). [file 41586_2024_7953_MOESM6_ESM.zip › DataS4/TmY9q/720575940610113604.pdf]

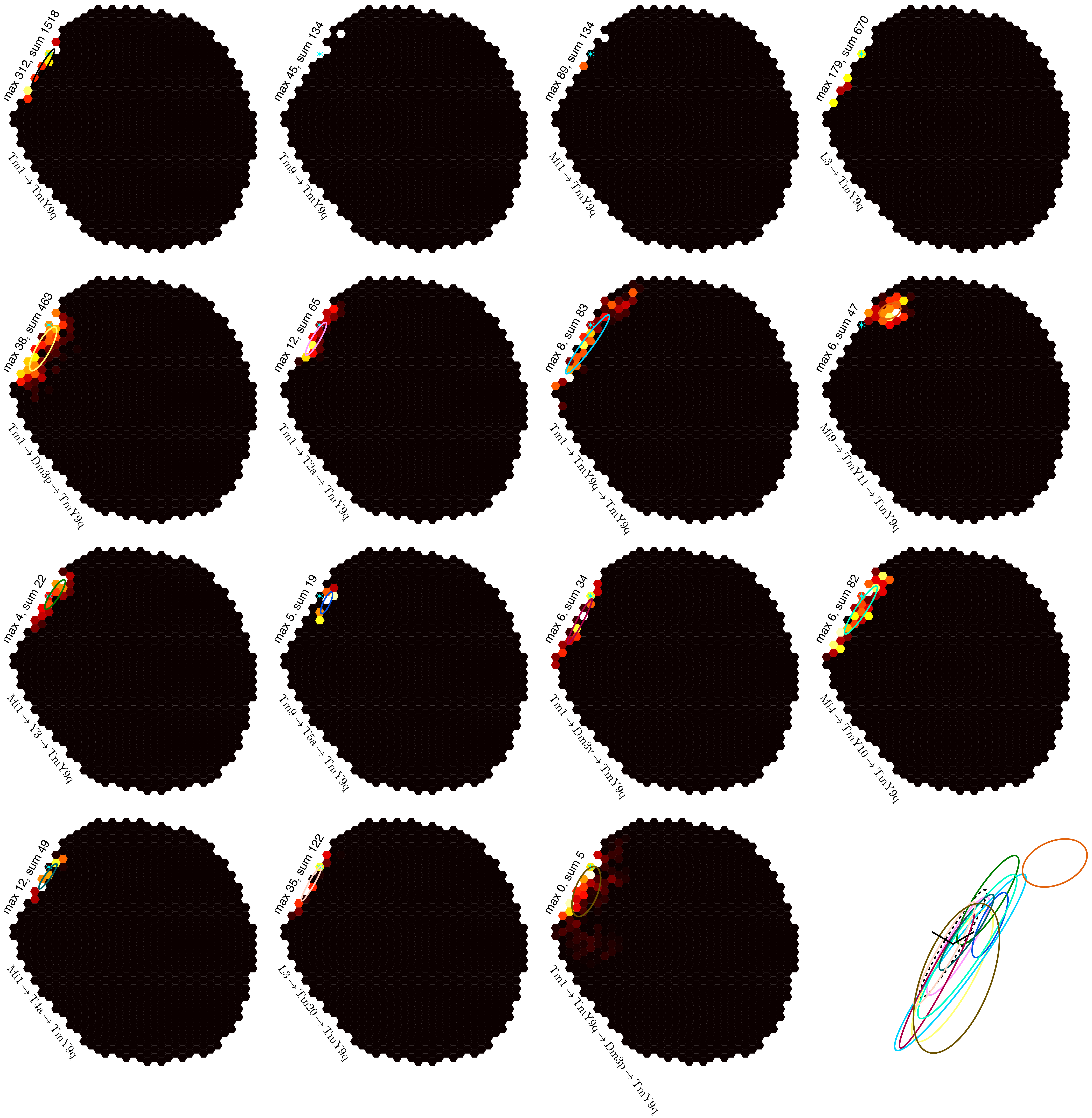
max 312, sum 1518
Tm1 → TmY9q
max 45, sum 134
Tm9 → TmY9q
max 89, sum 134
Mi1 → TmY9q
max 179, sum 670
L3 → TmY9q
max 38, sum 463
Tm1 → Dm3p → TmY9q
max 12, sum 65
Tm1 → T2a → TmY9q
max 8, sum 83
Tm1 → TmY9q → TmY9q
max 6, sum 47
Mi9 → TmY11 → TmY9q
max 4, sum 22
Mi1 → Y3 → TmY9q
max 5, sum 19
Tm9 → T5a → TmY9q
max 6, sum 34
Tm1 → Dm3v → TmY9q
max 6, sum 82
Mi4 → TmY10 → TmY9q
max 12, sum 49
Mi1 → T4a → TmY9q
max 35, sum 122
L3 → Tm20 → TmY9q
max 0, sum 5
Tm1 → TmY9q → Dm3p → TmY9q
